# Ultrasound-assisted immersion freezing method as a new strategy to improve the quality and microstructure of Chinese beef stewed dishes

**DOI:** 10.1016/j.ultsonch.2025.107373

**Published:** 2025-05-02

**Authors:** Liye Cui, Hengxun Lin, Xiaojia Hu, Wenqiang Guan, Xia Li, Wei Jia, Yiping Yang, Yong Chen, Chunhui Zhang

**Affiliations:** a Institute of Food Science and Technology, Chinese Academy of Agricultural Sciences, Key Laboratory of Agro-products Quality and Safety Control in Storage and Transport Process, Ministry of Agriculture and Rural Affairs, Beijing 100193, China; b Tianjin Key Laboratory of Food Biotechnology, Tianjin University of Commerce, Tianjin 300134, China

**Keywords:** Prepared dish, Braised beef with potato, Ultrasound-assisted immersion freezing, Microstructure, Meat quality

## Abstract

Prepared dishes, as a technological extension of pre-conditioned foods and representative products of the central kitchen, are becoming a major part of people’s daily diets. However, the problem of dish quality deterioration owing to freezing in the frozen supply chain has not yet been addressed, and freezing quality enhancement techniques are lacking. Herein, special equipment was used to investigate effects of ultrasound-assisted immersion freezing (UIF) on the quality of the prepared dish (braised beef with potato) and compare it with immersion freezing (IF) and conventional air freezing (AF). Results indicated that UIF effectively shortened the freezing time and improved the freezing rate of the dish. A suitable intensity of UIF treatment (150 W) improved dish quality, and high-intensity UIF treatments (300 and 450 W) caused tissue structure destruction and decreased quality. The hardness of beef and potatoes treated with UIF-150 was significantly higher than that of other treatments (*P* < 0.05). Scanning electron microscopy demonstrated that UIF-150 reduced the size of ice crystals and maintained the structural integrity of beef and potatoes. Porosity values of beef were 6.91 %, 2.66 %, 2.09 %, 4.30 % and 6.94 % in the AF, IF, UIF-150, UIF-300 and UIF-450 groups, respectively, and the trend of porosity in potatoes was similar to that of beef. Meanwhile, the contents of volatile flavour components in the dishes were remarkably decreased after freezing treatments, and UIF treatment could promote the retention of volatile flavour components. Results of this study provided a reference for the application of UIF technology in the frozen quality enhancement of prepared dishes and their industrialisation.

## Highlights

• UIF effectively reduced the overall freezing time of dishes and increased the freezing rate.
• UIF enhanced volatile flavour substances of beef and potatoes in dishes.
• Low-intensity UIF improved dish quality, and high-intensity UIF destroyed the structure of tissues.
• UIF-150 could achieve better quality control during the freezing of braised beef with potato dishes.
• UIF-150 could decrease water migration and maintained the integrity of beef fibres and potato cell morphology.

## Graphical abstract

**Figure:**
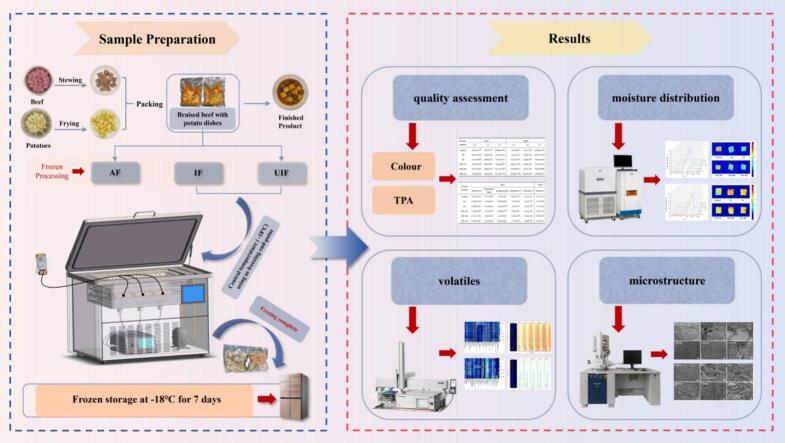

## 1 Introduction

Industrialised or prepared dishes are favoured by consumers for their cost-controllable, convenient and fast advantages, which highly meet the needs of the current fast-paced and diversified lifestyles [1,2]. In recent years, China’s prepared dish processing industry has developed rapidly. The market size reached $71.7 billion in 2023, and it is expected to reach $148.9 billion in 2026 [3]. The industrialised processing industry of Chinese dishes has a huge growth space for development. At present, freezing is the main mode of storage and transportation of prepared dish products. The cold chain is the foundation for the development of prepared dishes. It connects prepared dish producers with end consumers and determines the storage time and distribution distance of prepared dishes. However, owing to the complex nature of food systems in dishes, deterioration in colour, aroma, taste and shape quality can easily occur during the frozen storage process. Therefore, an effective preservation technology is necessary for the supply and circulation of prepared dishes.

Freezing is an effective method for preserving food quality and freshness, playing a vital role in maintaining the quality and safety of food and extending the shelf life of products by inhibiting the growth of microorganisms and biochemical reactions [4]. However, the formation of ice crystals during the freezing process can destroy the fibre and cell membrane structure of food, resulting in the deterioration of food quality during cold chain transportation and storage [5]. In the slow freezing process, large and uneven ice crystals are easily formed, which can increase the loss of food quality, resulting in poor overall structural stability, serious colour deterioration, a soft texture and accelerated oxidation [2,6]. According to statistics, the global loss of agricultural products during cold chain transportation and storage reached up to 50 % in 2023 [7]. Therefore, continuing to develop advanced technology to improve the quality of frozen prepared dishes is an effective way to reduce these losses.

Previous studies have reported that several novel freezing technologies, such as high-pressure freezing [8,9], electromagnetic field–assisted freezing [10,11], radio frequency–assisted freezing [12,13] and ultrasonic-assisted freezing [14,15], play an active role in improving the freezing rate and enhancing the quality of frozen food. Among them, ultrasonic-assisted immersion freezing (UIF), which uses a liquid medium as the coolant, has a faster freezing rate than other air-medium freezing technologies and is more suitable for the frozen processing of packaged food. Additionally, ultrasound is an emerging sustainable technology that has shown promising results in improving the rate of food freezing, promoting nucleation and reducing ice crystal size [16,17]. This is primarily attributed to the cavitation effect of ultrasound waves that produces cavitation bubbles in the transmission medium, and the movement of these cavitation bubbles creates a micro-fluidic effect, which can improve the heat transfer and mass transfer efficiency of the freezing process [18,19]. Furthermore, the cavitation bubbles can serve as the initial crystal nuclei, increasing the number of nucleation sites and promoting the formation of ice crystals [17]. Meanwhile, the micro-flow effect can break down larger ice clusters formed during crystal growth into smaller ice crystals, further reducing the size of the ice crystals [20,21].

In recent years, research on ultrasound-assisted freezing technology has primarily focused on the treatment of raw materials such as fresh poultry and livestock, aquatic products and agricultural products. Zhang et al. [22] investigated the effects of different ultrasonic powers on the freezing rate and quality of the pig longissimus muscle and found that the phase transition time of the sample at 180 W ultrasonic power was the shortest, with smaller and more evenly distributed ice crystals formed. Zhang et al. [23] indicated that ultrasonic-assisted freezing could effectively delay protein oxidation and structural damage during the frozen storage of chicken breasts, thereby improving the quality of frozen chicken breasts. Sun et al. [24] discovered that ultrasonic-assisted freezing slowed down the internal water migration in carp, reduced thawing and cooking losses and resulted in higher protein thermal stability. Tian et al. [25] reported that orthogonal ultrasound significantly reduced the drip loss and increased the hardness of potatoes by increasing the freezing rate and shortening the total freezing time. To preserve the quality attributes (e.g., color, texture, moisture retention) of multi-component prepared dishes during frozen storage, it is essential to develop advanced freezing technologies, such as ultrasound-assisted immersion freezing, which can minimize ice crystal damage and stabilize microstructure. Therefore, this study evaluated the feasibility and applicability of UIF in enhancing the quality of prepared dishes (braised beef with potato) in terms of colour, texture, flavour profile and microstructure, providing a theoretical basis for the development of a new type of cold chain technology for prepared dishes. This study fills a critical research gap in the application of UIF technology for cryopreservation of complex multi-ingredient dishes.

## 2 Materials and methods

### 2.1 Raw materials

Fresh beef tenderloin and Holland-15 potatoes were purchased from a local market (Shangdi Market in Beijing, China) as raw materials. The beef was immediately placed in an incubator with ice packs and sent to the laboratory under 4 °C. The potatoes were placed in a black plastic bag and delivered to the laboratory under 4 °C. The beef was cut into evenly sized cubes (average weight: 20.0 ± 5.0 g, size: 2 × 2 × 2 cm^3^) after removing the external connective tissue and fat and the potatoes were peeled and cut into cubes (average weight: 30.0 ± 5.0 g, size: 2.5 × 2.5 × 2.5 cm^3^) for later use. A braised beef with potato dish was prepared using the method reported in our previous study [26]. The beef was subjected to tumbling and pickling prior to stewing, while the potatoes were immersed in saline solution (1.0 % NaCl, w/v) and subsequently deep-fried. After cooling, the stewed beef, fried potatoes, and soup were combined at a mass ratio of 1:2:2 (beef: potatoes: soup). The final product (total weight: 220 ± 15 g) was vacuum-sealed in a retort pouch (15 cm × 20 cm, 20 μm thickness; Beijing Shoutian Zhixin Technology Co., Ltd., Beijing, China) using a vacuum packaging machine (ZK-420; Quanzhou Huapai electromechanical Co., LTD, Fujian, China) under a vacuum pressure of 0.08 MPa for 8 s.

### 2.2 Freezing process

**Fig. 1:**
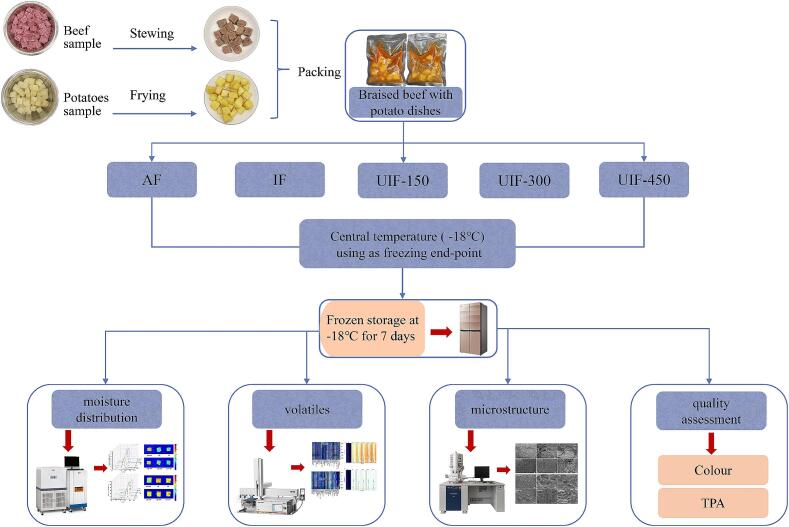
Flow chart for experiment design.

**Fig. 2:**
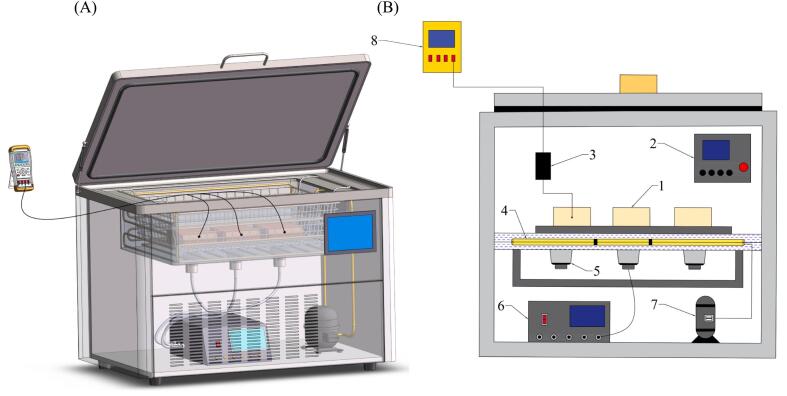
Schematic of the ultrasound-assisted immersion freezing equipment (A) and internal structure of the device (B). 1. Frozen samples. 2. Total control panel. 3. Temperature sensor. 4. Condensator. 5. Ultrasonic transducer. 6. Ultrasonic generator. 7. Refrigeration compressor. 8. Temperature recorder.

The packed dishes were frozen using five different methods: air freezing (AF), immersion freezing (IF) and UIF at power levels of 150  W, 300  W, and 450  W (Fig. 1). The AF samples were frozen in a commercial refrigerator at −20.0 ± 0.5 °C until the centre temperature of the beef and potatoes reached −18 ± 0.5 °C. The UIF and IF samples were treated using an integrated ultrasonic immersion freezer, as shown in Fig. 2. The equipment was designed and manufactured by our team, which allows for the adjustment of ultrasonic frequency, power, time and other parameters. The refrigeration tank with a constant low temperature in the equipment maintained the freezing temperature at −20.0 ± 0.5 °C, and 95 % ethanol was used as the coolant. The output power of the ultrasonic device was set to 0 W (IF), 150 W (UIF-150), 300 W (UIF-300) and 450 W (UIF-450) with a frequency of 40 kHz. The temperature of the samples was recorded using a T-type thermocouple (ST1008, Gathering International High-tech Equipment Co., Ltd., Hangzhou, China). When the centre temperature of the samples reached 0 °C, the ultrasonic equipment was turned on, then turned off the equipment until the terminal temperature of samples reached −18 ± 0.5 °C. The frozen samples were then transferred to a commercial refrigerator (BCD-520WQ3S, Changhong Meiling Co., Ltd., Beijing, China) and stored for 7 days. Subsequently, the frozen samples were reheated in a boiling water bath (DK-8D, Xinrui Instrument Co., Ltd., Jiangsu, China) until the central temperature of the beef and potatoes reached 72.0 ± 0.5 °C for further quality tests.

### 2.3 Freezing curve

The freezing curve was determined using the correction method reported by Yu et al. [27]. A multi-channel temperature measuring instrument (ST1008, Gathering International Equipment Co., Ltd., Hangzhou, China) probe was inserted into the centre of the beef and potato samples to record the temperature every 10 s until the sample centre temperature reached −18 °C. Then, the freezing curve was plotted.

### 2.4 Quality assessment

#### 2.4.1 Colour attributes

Colour attributes were measured according to the method described by Li et al. [26]. The measurement was performed using a portable colorimeter (CR-400, Minolta, Osaka, Japan), which was calibrated using a white tile before testing. The liquid on the surface of the beef and potato cubes was wiped with filter paper, and the samples were placed in the lens port of the colorimeter and spread at the bottom of the colorimeter for colorimetric measurements. Each sample was rotated six times in the same direction. The brightness (L*), redness (a*) and yellowness (b*) values of the sample were recorded. According to the study by Holman et al. [28] and Manzocco et al. [12], the chroma value, hue angle (*h°*) and color differences value (ΔE*) were calculated with the following formulas:

(1) $$Chroma=\sqrt{{a*}^{2}+{b*}^{2}}$$

(2) $$Hue\;angle=arctan\left[b^{*}/a^{*}\right]$$

(3) $$\Delta E=\sqrt{{\left(L^{*}-L_{0}^{*}\right)}^{2}+{\left(a^{*}-a_{0}^{*}\right)}^{2}+{\left(b^{*}-b_{0}^{*}\right)}^{2}}$$

where L_0_*, a_0_*, and b_0_* were the color parameters of unfrozen samples.

#### 2.4.2 Texture profile analysis (TPA)

The texture of beef was determined according to the protocol described by Luo et al. [29] with slight modifications. The thawed beef was cut into 1 × 1 × 1 cm^3^ pieces along the direction of the muscle fibres and measured the texture property using a TPA texture analyser (TA-XT plus, Stable Micro-Systems, Surrey, UK) equipped with a P/36 R flat cylindrical probe. The parameters were set as follows: trigger force of 5.0 g, compression ratio of 40 % and constant speeds of 2.0 mm/s (pre-test), 1.0 mm/s (test) and 2.0 mm/s (post-test), with 5 s intervals between compression cycles.

The hardness of the potatoes was measured according to the method reported by Li et al. [26]. The parameters were set as follows: compression ratio of 60 % and 5.0 mm/s (post-test). The probe type, trigger force, pre-test and test speed parameters were consistent with those of beef.

### 2.5 Moisture state and distribution analysis

Magnetic resonance imaging (MRI) and low-field nuclear magnetic resonance (LF-NMR) were used to determine the water distribution and migration of the beef and potato samples according to the modified method reported by Lin et al. [30]. Water state was measured using an LF-NMR analyser (MesoMR23-060H, Niu Mark Company, Shanghai, China). The samples were cut into 2 × 2 × 2 cm^3^ chunks and placed in the middle of a 60-mm diameter tube. Carr–Purcell–Meiboom-Gill sequence (CPMG) was used to measure the transverse T_2_ relaxation time of the sample. The sampling parameters of beef were as follows: SW = 200 KHz, TW = 1500 ms, TE = 0.5 ms, NECH = 5000 and NS = 4. The pulse widths at 90° and 180° were 200 μs. The sampling parameters of potatoes were as follows: SW = 200 KHz, TW = 3500 ms, TE = 0.2 ms, NECH = 5000 and NS = 4. The pulse widths at 90° and 180° were 200 μs.

MRI scans of the samples were recorded using a MINI MR-60 instrument (Niumag Electric Company, Shanghai, China) (spectrometer frequency: 23.292 MHz; operating temperature: 32.0 °C). Proton-density images were acquired using the MSE imaging sequence with a repeat time (TR) of 500 ms and an echo time (TE) of 20 ms. The samples were divided into three layers for analysis, each with a width of 2.0 mm and a slice gap of 1.7 mm, and the observation position was the cross profile. The grayscale image was processed with pseudo-colour using image processing computer software to analyse the hydrogen proton-density intensity.

### 2.6 Volatile flavour compounds identification

GC–IMS analysis was performed as described by Sun et al. [31] with a slight improvement. Here, 2 g each of beef and potato samples was taken and incubated at 65 °C for 15 min in 20 mL headspace injection bottles. The injection parameters were set as follows: injection temperature of 75 °C, incubation shake bottle speed of 500 r/min and injection volume of 0.5 mL. The relevant GC parameters were as follows: MXT-5 column (15 mL, 0.53 mm ID and 1 μm FTK) was used, the column temperature was maintained at 60 °C, the carrier gas was high-purity N_2_ (99.99 % purity) and the running time was 30 min. The initial flow rate of the carrier gas was maintained at 2 mL/min for 2 min and then increased linearly to 10 mL/min over 8 min, to 100 mL/min over the next 10 min, and finally, to 150 mL/min over the last 10 min. The relevant parameters of the ion migration spectrum were set as follows: drift tube temperature of 45 °C, positive-ion mode of ionisation and drift gas flow rate of 75 mL/min.

### 2.7 Microstructure

#### 2.7.1 Scanning electron microscopy (SEM)

SEM of beef samples was conducted using the modified method reported by Cheng et al. [32]. The samples were cut into 4 × 3 × 2 mm^3^ pieces, fixed with 2.5 % glutaraldehyde solution for 24 h and then washed with distilled water. The samples were eluted at 0 %, 50 %, 70 %, 80 %, 90 %, 95 % and 100 % alcohol gradients and dehydrated for 15 min. Then, the samples were lyophilised and coated with palladium (Bal-TEC, Manchester, NH, USA) for observation.

According to the method reported by Zhou et al. [33], after different freezing treatments, the potato samples were freeze-dried in a freezer-dryer (FD-1A-50, Beijing Boyuan Kang Experimental Instrument Co., Ltd., Beijing, China). The samples were then cut into 4 × 4 × 2 mm^3^ cubes using Leica blades and fixed in the special metal sample table and coated using a gold–palladium alloy coater. The sample table was then pushed into the chamber of the SEM instrument (SU8010, Hitachi, Tokyo, Japan) and vacuumed. Surface morphologies of the beef and potato samples were observed at 300-fold and 50-fold magnification, respectively.

#### 2.7.2 Transmission electron microscopy (TEM)

TEM analyses of the beef and potato samples were conducted using the modified methods reported by Lin et al. [2] and Zhang et al. [34], respectively. The beef and potato samples were cut into strips (5 × 3 × 2 mm^3^) and then fixed in 2.5 % and 3.5 % glutaraldehyde solution for 24 h, respectively, during which time the solution was constantly changed until it became clear. The samples were washed thrice with 0.2 M phosphate buffer (pH 7.4) for 15 min each time and then post-fixed with 1 % osmium tetroxide for 2 h. The fixed strip samples were then cleaned with 0.2 M phosphate buffer (pH 7.4) and ethanol gradient solutions (30 %, 50 %, 70 %, 80 %, 90 %, 95 % and 100 %; 15 min at a time) to dehydrate. Ethanol acetone replacement was used, and then, pure resin (812#) was used for embedding. Then, the strips were cut into ultrathin sections (80 nm thickness) with a cryostat microtome (Leica EM UC7), and the beef and potato sample slices were stained with uranyl acetate and lead citrate for 15 min. Finally, visual images of the beef and potato sample slices were captured using TEM (HT7800, Hitachi, Tokyo, Japan) at magnification levels of ×12000 and ×3000, respectively.

### 2.8 Statistical analysis

The significance of the differences was evaluated using one-way analysis of variance. Data were presented as mean ± standard deviation and analysed using SPSS 22.0 software (IBM Corporation, Armonk NY, USA). Graphs were generated using Origin 9.1 software. Vocal and its plugins (Reporter, Gallery plot) analysis software was used to analyse volatile flavour substances, and the GC–IMS Library was used to characterise compounds. Image J software (National Institutes of Health, Bethesda, Maryland, USA) was used to outline each pore in the SEM image and calculate the area to determine the porosity size of the frozen sample.

## 3 Results and discussion

### 3.1 Effect of different freezing methods on the freezing rate

**Fig. 3:**
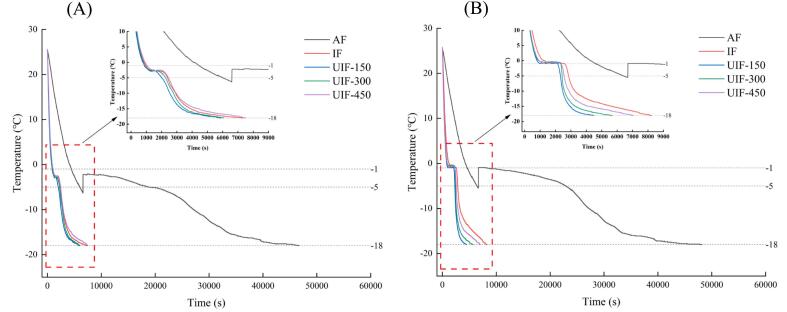
Freezing curves of beef (A) and potatoes (B) in braised beef with potato dishes under different freezing treatments. AF, air freezing; IF, immersion freezing; UIF, ultrasound-assisted immersion freezing at different ultrasound powers (150, 300 and 450 W).

The freezing rate significantly affects the quality of frozen food. During the freezing process, a faster rate tends to produce fine and more uniform ice crystals, thereby minimising damage to the internal cells, tissues and proteins of the food, which helps in maintaining the nutritional value and quality of the food [22]. The temperature curve directly reflects the change characteristic of the freezing process. Freezing curves and freezing time of beef and potatoes are shown in Fig. 3 and Table 1, respectively. Compared with AF, IF significantly shortened the freezing time for the samples (*P* < 0.05), and UIF overall enhanced the freezing rate of beef and potato samples. The overall freezing time of beef and potatoes at 150 W ultrasonic power was significantly shorter than that of other treatments (*P* < 0.05), with reductions of 87.86 % and 90.28 %, respectively, compared with AF. This superior freezing effect may be because ultrasound promoted the formation of crystal nuclei and enhanced heat transfer efficiency.

**Table 1:** Freezing parameters of beef and potatoes under different freezing treatments.

Freezing methods	Beef		Potato	
	Total freezing time (s)	Phase transition stage (s)	Total freezing time (s)	Phase transition stage (s)
AF	46 075.00 ± 1 778.57^a^	14 533.50 ± 984.17^a^	48 929.50 ± 1 225.08^a^	23 131.50 ± 1 952.24^a^
IF	7 120.00 ± 175.69^c^	1 500.00 ± 46.90^b^	8 240.00 ± 54.77^b^	1 597.50 ± 55.60^b^
UIF-150	5 950.00 ± 163.71^e^	1 297.50 ± 109.05^c^	4 757.50 ± 197.72^e^	1 140.00 ± 59.44^e^
UIF-300	6 700.00 ± 118.32^d^	1 472.50 ± 34.03^b^	5 790.00 ± 95.57^d^	1 377.50 ± 37.75^d^
UIF-450	7 352.50 ± 126.06^b^	1 555.00 ± 28.87^b^	6 945.00 ± 208.25^c^	1 492.50 ± 30.96^c^

The means in the same column with different superscript letters differ significantly (*P* < 0.05). AF, air freezing; IF, immersion freezing; UIF, ultrasound-assisted immersion freezing at different ultrasound powers (150, 300 and 450 W).

In the process of ultrasonic propagation, a large number of cavitation bubbles are formed, grown, oscillated and then broken [35]. The micro-flow generated by the movement of the cavitation bubbles causes violent collisions between particles in the medium, resulting in strong turbulence that aids in inducing nucleation and enhancing heat and mass transfer efficiency [18]. Moreover, the cavitation bubbles serve as the initial crystal nucleus, promoting the formation of ice crystals. When the bubbles burst, a micro-jet will be formed, which affects the dendritic crystal at the end, forming a large number of crystal fragments, which can be used as a crystal nucleus to induce the formation of more ice crystals [23]. Following the bubbles’ collapse, the resulting high pressure increases the sample’s equilibrium freezing temperature and the degree of supercooling, providing the driving force for ice crystal formation [36]. Consequently, UIF can induce nucleation, improve heat and mass transfer efficiency and reduce the freezing time.

The period at the temperature range of −1 °C–−5 °C is the maximum stage of ice crystal formation, also known as the phase transition stage, which affects the size and distribution of the ice crystals during freezing [37]. The shorter the duration of this maximum ice crystal formation stage is, the smaller and more evenly distributed are the ice crystals and the lower is the degree of food quality damage [38]. As shown in Table 1, the phase transition time of beef and potatoes in the UIF-150 group was the shortest. Compared with AF and IF, the phase transition time of beef was shortened by 91.07 % and 13.50 %, respectively, and that of potatoes was shortened by 95.72 % and 28.64 %, respectively. However, excessive ultrasonic power also produced negative effects on the freezing rate of the dishes. As shown in Fig. 3, with an increase in ultrasonic power, the phase transition time of beef and potatoes lengthened and the freezing rate decreased. This could be attributed to the formation of too many cavitation bubbles and the generation of excess heat, which cannot be dissipated in a timely manner, resulting in heat transfer efficiency and freezing rate decrease, as well as an increase in phase transition time, consistent with the results reported by Zhang et al. [39].

### 3.2 Effect of different freezing methods on quality

#### 3.2.1 Colour value

Colour appearance is a crucial indicator evaluating the quality of food products and determines the overall acceptability. As shown in Table 2, the L* value of beef in the AF group was the highest, significantly higher than that in other freezing groups (*P* < 0.05). This can be attributed to the slow freezing rate, which leads to the formation of large and uneven ice crystals, causing increased tissue damage to the beef and allowing free water to migrate to the surface, thereby reflecting more light [40]. No significant differences were observed in the a* value and of beef across different ultrasound groups (*P* > 0.05). As ultrasound power was increased, the L* value and b* value of beef exhibited a trend of initially decreasing before increasing. Meanwhile, the AF-treated beef demonstrated the largest color difference (ΔE*) and the smallest chroma values. UIF treatment showed negligible impact on both hue angle of beef samples (*P* > 0.05). Compared with the unfrozen samples, the L* value of potatoes after different freezing treatments was significantly decreased (*P* < 0.05). The L* value of potatoes in the UIF-150 treatment was the highest, resembling that of the unfrozen sample. The L* value of potatoes in the AF group was the lowest and a* value was the highest, which could be attributed to the extensive damage to the texture of potatoes by the large ice crystals generated. The soup was easy to adsorb on the surface of potatoes, resulting in the darkening of the overall colour and redness value. Additionally, the AF group exhibited a significantly higher *h°* value compared to other treatment groups (*P* < 0.05), indicating a distinct shift toward red-dominated hue characteristics. Following ultrasound treatment, the b* values of potatoes were significantly higher than those in the control group (*P* < 0.05), and the a* and b* values were the largest in the UIF-150 group. After freezing treatment, the color difference (ΔE*) of potatoes ranged from 4.22 to 11.58, corresponding to perceptible visual differences. Notably, the AF group exhibited the largest ΔE* value, while the UIF-150 group showed significantly lower ΔE* compared to other ultrasonic treatment groups, demonstrating stable color characteristics.

**Table 2:** Influence of different freezing methods on the color of braised beef with potato dishes.

Freezing	Beef						Potato					
	L*	a*	b*	C*	*h°*	ΔE*	L*	a*	b*	C*	*h°*	ΔE*
Control		2.81 ± 0.23^b^	10.88 ± 0.50^b^	11.15 ± 0.42^ab^	14.52 ± 0.68^cd^		50.10 ± 0.95^a^	−2.13 ± 0.74^e^	18.83 ± 2.04^bc^	21.17 ± 0.42^a^	354.31 ± 1.41^a^	
AF	38.65 ± 0.79^a^	3.45 ± 0.14^a^	10.21 ± 0.41^c^	10.20 ± 0.39^d^	18.37 ± 0.83^a^	5.31 ± 0.48^a^	41.10 ± 2.13^e^	5.02 ± 0.78^a^	18.97 ± 1.15^bc^	18.68 ± 0.39^b^	15.69 ± 1.32^b^	11.58 ± 1.44^a^
IF	35.85 ± 0.86^b^	3.00 ± 0.21^a^	9.74 ± 0.55^d^	11.57 ± 0.50^a^	17.39 ± 0.45^b^	2.90 ± 0.17^b^	44.16 ± 1.91^d^	2.26 ± 0.19^b^	18.54 ± 0.98^c^	19.25 ± 0.21^b^	9.92 ± 1.09^c^	6.60 ± 0.58^b^
UIF-150	34.30 ± 1.60^c^	2.96 ± 0.38^b^	11.64 ± 0.69^a^	10.82 ± 30.31^bc^	13.75 ± 0.68^d^	1.56 ± 0.36^c^	48.63 ± 1.33^b^	1.26 ± 0.28^c^	21.36 ± 1.51^a^	20.78 ± 1.03^a^	3.62 ± 0.61^d^	4.22 ± 0.53^d^
UIF-300	32.27 ± 0.76^e^	2.86 ± 0.23^b^	10.73 ± 0.50^b^	11.39 ± 0.47^a^	14.97 ± 0.53^c^	1.20 ± 0.27^c^	45.89 ± 0.87^c^	0.21 ± 1.12^d^	19.61 ± 0.88^bc^	19.28 ± 0.49^b^	3.20 ± 0.63^d^	5.24 ± 0.29^cd^
UIF-450	34.03 ± 0.78^d^	2.81 ± 0.21^b^	11.18 ± 0.55^b^	10.66 ± 0.40^c^	13.93 ± 0.59^d^	1.48 ± 0.12^c^	45.29 ± 1.79^cd^	0.52 ± 0.97^cd^	19.83 ± 0.85^b^	19.21 ± 0.53^b^	3.22 ± 1.57^d^	5.85 ± 0.55^bc^

The means in the same column with different superscript letters differ significantly (*P* < 0.05). AF, air freezing; IF, immersion freezing; UIF, ultrasound-assisted immersion freezing at different ultrasound powers (150, 300 and 450 W).

#### 3.2.2 TPA analysis

The change in texture is an essential indicator for evaluating dish quality. As shown in Table 3, the hardness of beef and potatoes in the frozen dishes decreased and was significantly lower than that of the control group (*P* < 0.05). The hardness and chewiness values of beef in the AF group were 14.69 and 2.13 N, respectively, which decreased by 26.73 % and 38.26 % compared with those of unfrozen samples. This could be attributed to the formation of large ice crystals during freezing, increasing the damage to the muscle fibre structure of beef [41]. Compared with the control group, the hardness values of potatoes in the AF and IF groups were reduced by 84.32 % and 77.71 %, respectively, considerably affecting the texture and taste of potatoes. This result could be attributed to the destruction of potato cell and tissue structure by ice crystals. Tian et al. [42] demonstrated that the formation of ice crystals during the freezing process led to the separation, destruction and deformation of radish cells. UIF improved texture retention, with the UIF-150 group exhibiting significantly higher hardness and chewiness values for beef compared with other treatment groups (*P* < 0.05), resembling those of the unfrozen samples. The potato samples in the UIF-150 group achieved the highest hardness value of 4.67 N, a 49.25 % increase over the AF group, thereby preserving the texture more effectively. This improvement can be attributed to the ultrasonic treatment, which accelerated the freezing rate of the dishes, reduced the phase transition time, fostered the development of fine and uniform ice crystals, minimised the damage to beef tissue fibres caused by ice crystal formation and ultimately sustained the overall dish quality through cavitation and mechanical action [43]. As the ultrasonic power increased, the hardness and chewiness of beef gradually decreased. In the UIF-450 group, the hardness and chewiness values of beef were 13.81 and 2.18 N, respectively, which were 22.98 % and 32.09 % lower compared with those in the UIF-150 group. Meanwhile, the hardness value of potatoes was 2.03 N, which was significantly lower than that of other ultrasonic groups (*P* < 0.05). This difference could be attributed to the excessive ultrasonic power resulted in a longer time to pass through the phase transition stage, which was easy to form large ice crystals, increased the destruction of ice crystals to beef fibre structure. In addition, the excessive formation of cavitation bubbles led to the generation of strong shock waves when the bubbles burst, further increasing the damage to the beef tissue fibres and potato cell structure [39].

**Table 3:** Effects of different freezing methods on texture characteristics of braised beef with potato dishes.

Freezing methods	Beef					Potato
	Hardness/N	Cohesiveness/Ratio	springiness/mm	Gumminess/N	Chewiness/N	Hardness/N
Control	20.05 ± 2.84^a^	0.37 ± 0.02^d^	1.13 ± 0.18^a^	6.94 ± 0.33^b^	3.45 ± 0.12^a^	15.12 ± 1.35^a^
AF	14.69 ± 1.53^cd^	0.41 ± 0.02^c^	0.98 ± 0.09^bc^	7.83 ± 0.88^a^	2.13 ± 0.67^b^	2.37 ± 0.30^de^
IF	15.90 ± 0.92^c^	0.42 ± 0.02^c^	1.02 ± 0.05^abc^	8.20 ± 0.93^a^	2.12 ± 0.28^b^	3.37 ± 0.26^c^
UIF-150	17.93 ± 0.93^b^	0.47 ± 0.03^a^	1.08 ± 0.11^ab^	7.94 ± 0.85^a^	3.21 ± 0.25^a^	4.67 ± 0.74^b^
UIF-300	15.87 ± 1.28^c^	0.44 ± 0.02^b^	0.93 ± 0.06^c^	7.56 ± 0.49^ab^	2.36 ± 0.26^b^	2.92 ± 0.60^cd^
UIF-450	13.81 ± 0.86^d^	0.44 ± 0.03^b^	0.91 ± 0.08^c^	7.72 ± 0.59^a^	2.18 ± 0.35^b^	2.03 ± 0.13^e^

The means in the same column with different superscript letters differ significantly (*P* < 0.05). AF, air freezing; IF, immersion freezing; UIF, ultrasound-assisted immersion freezing at different ultrasound powers (150, 300 and 450 W).

### 3.3 Effect of different UIF treatments on moisture state and distribution

**Fig. 4:**
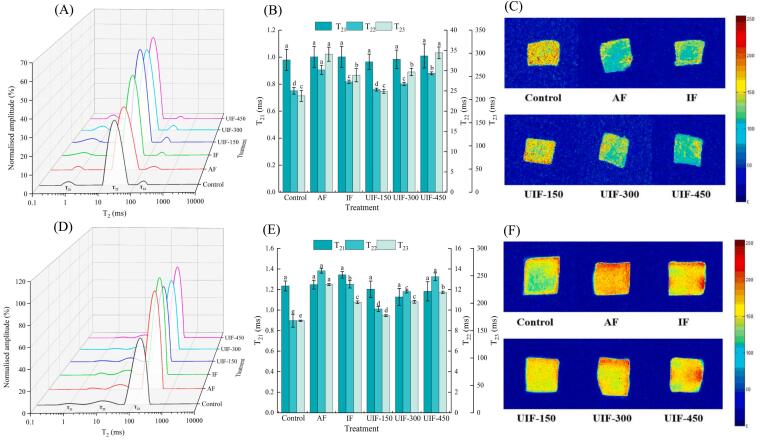
Water distribution, T_2_ relaxation time and magnetic resonance images of beef (A–C) and potatoes (D–F) in braised beef with potato dishes under different freezing treatments. The means in the same T_2_ with different lowercase letters (a–e) differ significantly (*P* < 0.05). AF, air freezing; IF, immersion freezing; UIF, ultrasound-assisted immersion freezing at different ultrasound powers (150, 300 and 450 W).

LF-NMR technology has been widely used to characterise the changes of bound, fixed and free water in food processing [44]. Effects of different freezing methods on the water states of beef and potatoes are shown in Fig. 4A and B, respectively. Three water peaks were identified in the T_2_ relaxation time curves of all samples: T_21_ (0–10 ms), T_22_ (10–100 ms) and T_23_ (100–1000 ms). These peaks represent bound water (beef: water molecules in protein structural gaps or binding to protein side chains and other macromolecules; potatoes: water bound to the interior of starch granules or to polysaccharides in the cell wall), water that does not flow easily (beef: water in the dense network of intracellular myofibrillar protein structures or in the cellular interstitial space; potatoes: poorly flowable water in the cell cytoplasm) and free water, respectively [44,45].

As shown in Fig. 4C and D, different freezing treatments had no significant effect on T_21_ relaxation time of beef and potatoes (*P* > 0.05). This might be because the bound water was very stable and tightly bound to large molecules such as proteins and polysaccharides and did not migrate easily [46]. During different freezing processes, the states of fixed water and free water undergo significant changes. The T_22_ and T_23_ relaxation times of frozen samples were significantly longer than those of the control group (*P* < 0.05), indicating that the freezing process reduced the stability of fixed water and free water in beef, leading to water migration and loss. Qian et al. indicated that the formation of large ice crystals during freezing could cause serious mechanical damage to the muscle structure, resulting in a high degree of denaturation of the muscle proteins and decreased ability to reabsorb water, thus affecting the relaxation behaviour of the water components [47]. As shown in Fig. 4D, the results of the moisture distribution of potatoes were similar to those of beef. The T_23_ relaxation time and free water content of potatoes increased significantly after freezing, which could be attributed to the damage of the potato cell membrane and tissue structure caused by freezing, resulting in internal water migration and external soup entering or adsorbing on the surface, and T_23_ relaxation time increased [24,48]. UIF treatment effectively slowed down the migration of water in the samples. The T_22_ and T_23_ relaxation times of beef in the UIF-150 group were significantly shorter than those in the AF group (*P* < 0.05), with reductions of 4.85 and 80.16 ms, respectively. These results could be attributed to the fact that ultrasonication increased the freezing rate of the samples, promoted nucleation of water molecules, accelerated the condensation of ice crystals and reduced the damage of ice crystals to the cell structure, thereby slowing down the migration of water [49]. Zhang et al. [50] reported that UIF-180 treatment led to the formation of smaller and more uniformly distributed ice crystals during storage, which helped in maintaining the structural integrity of pig longissimus muscle tissues and effectively slowed down free water migration. However, excessive ultrasonic power promoted water migration during the sample freezing process. The study revealed that no significant difference in the free water relaxation time between the UIF-450 and AF groups (*P* > 0.05) was observed, which could be attributed to the excessive ultrasonic power that reduced the freezing rate and exacerbated the destruction of the tissue by ice crystals [51].

MRI is widely used to visualise changes in water and water retention in food [52]. Fig. 4E and F show MRI images of samples with different freezing treatments, where the red areas indicate high hydrogen proton density and high water content and the blue areas indicate low hydrogen proton density and low water content [32]. As shown in Fig. 4E, beef in the control group had a high water content, uniform water distribution and large coverage of the red area. After different freezing treatments, the red region of the sample was significantly reduced and the distribution was uneven, indicating that freezing can lead to different degrees of water loss. Compared with other freezing treatments, the hydrogen proton density of beef in the UIF-150 treatment was higher and the water distribution was uniform, which might be attributed to the significantly increased freezing rate of beef under this condition and the formation of small and uniform ice crystals, which highly reduced the damage caused by ice crystals to the beef muscle fibre structure and maintained the ability of muscle fibres to fix water [53]. Wei et al. reported similar results, finding that the freezing rate of shrimp decreased as freezing temperatures increased, leading to the production of larger ice crystals that disrupted protein binding to water, resulting in increased water loss [37]. Fig. 4F shows the hydrogen proton density of the potatoes, indicating that the change in internal moisture in the potatoes exhibited an opposite trend to that observed in the beef. Compared with the control group, the hydrogen proton density of the frozen potatoes was higher, which could be attributed to the serious texture damage of the frozen potatoes. This damage may have facilitated the absorption of soup into the surface or interior of the potatoes, thereby increasing their water content and consequently elevating the hydrogen proton density. As the ultrasonic power increased, the hydrogen proton density of potatoes also increased, indicating that ultrasonic treatment can promote the water adsorption process of potatoes following freezing to some degree.

### 3.4 Effect of different UIF treatments on the volatile flavour compounds

**Fig. 5:**
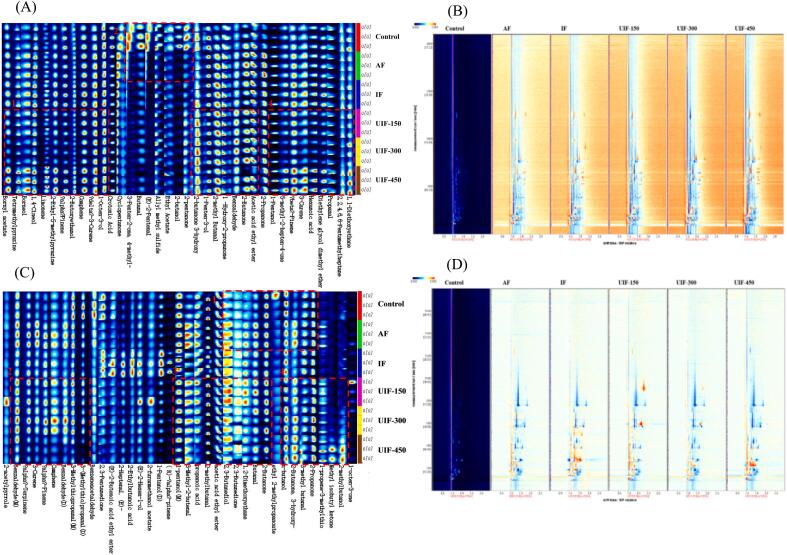
Fingerprint of volatile compounds and two-dimensional map of volatile compound of beef (A and B) and potatoes (C and D) in braised beef with potato dishes under different freezing treatments. AF, air freezing; IF, immersion freezing; UIF, ultrasound-assisted immersion freezing at different ultrasound powers (150, 300 and 450 W). “M” and “D” denote monomer and dimer, respectively.

Herein, 37 and 36 volatile flavour compounds were identified by GC–IMS in beef and potatoes, respectively, including 4 aldehydes, 6 alcohols, 9 hydrocarbons, 7 ketones, 2 acids, 3 esters, 1 phenol, 3 ethers, and 2 nitrogen-containing compounds in beef, and 5 aldehydes, 6 alcohols, 11 hydrocarbons, 7 ketones, 2 acids, 3 esters, and 1 nitrogen-containing compound in potatoes. Two-dimensional spectroscopy was used to compare the differences in volatile flavour substances in beef and potatoes after different freezing treatments. As shown in Fig. 5B and D, the horizontal coordinate indicates the ion migration time, the vertical coordinate indicates the retention time of gas chromatography and the red line parallel to the vertical coordinate at 1.0 is the reactive ion peak (RIP). Each data point appearing on both sides of the RIP peak represents a volatile compound, and the magnitude of the concentration can be indicated by the colours, where red indicates a high content of the compound and blue indicates a low content [54]. The volatiles determined using the GC–IMS technique had an excellent separation effect, and the differences between different freezing treatment groups were evident, primarily in the time, position, number and intensity of the ion peaks.

Fig. 5A and C show the fingerprints of the volatile flavour compounds of beef and potatoes under different freezing treatments, respectively. The compounds were divided into four regions, indicated by red boxes. Obvious differences in the volatile flavour compounds between beef and potatoes were observed. As shown in Fig. 5A, the beef fingerprint analysis revealed an increase in the content of aroma compounds such as δ-3-carene, camphene, limonene, 1-octen-3-ol, 2-butoxyethanol, 5-methyl-2-ethylpyrazine, 1,4-cineol, tetramethylpyrazine, and borneol in region 1, as well as volatile compounds, including 1,1-diethoxyethane, propanal, hexanoic acid, β-pinene, 5-methyl-2-hepten-4-one, and 1-pentanol, in region 4 after freezing treatment. These results might be attributed to the fact that freezing causes damage to the beef’s texture, enlarging the pore space between fibres, and the soup could easily enter or adsorb on the surface, thereby enhancing the content of flavour substances. Notably, the concentration of 5-methyl-2-ethylpyrazine, a Maillard reaction product characterized by a roasted aroma, was markedly higher under UIF-450 treatment. This suggests that UIF not only preserves the aroma but also promotes the Maillard reaction, thereby achieving an aroma-enhancing effect. In region 3, the content of aroma compounds including 2-butanone, 1-hydroxy-2-propanone, acetic acid ethyl ester and 2-butanone 3-hydroxy, were markedly increased after ultrasonic treatment, which could be attributed to the promotion of mass transfer by ultrasound. Cárcel et al. [55] demonstrated that during the process of ultrasonic-assisted pork fillet curing, the moisture and NaCl contents in the sample increased as the ultrasonic power increased and ultrasound improved the internal and external mass transfer. Furthermore, it might be because ultrasound significantly improved the freezing rate of dishes, and a high freezing rate can retain more volatile flavour substances [29]. After freezing, the contents of volatile flavour compounds such as 2-pentanone, 2-butanol, ethyl acetate, (E)-2-pentenal and butanal in region 2 decreased, which might be attributed to the fact that freezing promoted water migration in beef and led to the dissolution and release of volatile substances, which resulted in a decrease in their content. Luo et al. [29] reported that the increased water loss after freezing could lead to a decrease in the retention rate and content of volatile substances.

Meanwhile, the substantial damage of freezing to the texture and composition of dishes may affect the retention of volatile flavour substances. Studies have shown that different protein fractions have different adsorption and binding capacities for flavour substances and that these capacities are closely related to protein structure and properties [56,57]. Wang et al. [58] found that within 5 min after heating, proteins would unfold, causing exposure of hydrophobic groups and promoting binding with aromatic compounds through hydrophobic interactions. Additionally, the interaction between protein, water and lipid molecules can form a high-density and stable network structure that inhibits the movement of water and macromolecules [59]. The ice crystals formed in the freezing process can break the stable network structure, increase protein denaturation and change the molecular properties and structure of proteins, leading to a decrease in the proteins’ ability to bind flavour compounds and the release of a large number of volatile flavour compounds [59,60].

Fig. 5C shows the potato fingerprint, indicating that the change in volatile flavour compound content in potatoes is similar to that in beef. Compared with the control group, the contents of 2-propanone, 3-hydroxy-2-butanone, 2-Propanone, butanal, and 2,3-butanedione in region 1 increased after freezing. The UIF-150 treatment promoted the retention of aroma compounds, including 2-butanone, 2,3-butanedione, 2,3-butanediol and 2-methylbutanal, in region 3, and the content increased significantly following UIF-150 treatment. In region 4, the contents of volatile compounds such as 2-propanone, 3-hydroxyl-2-butanone, and n-butanol increased with an increase in ultrasonic power. High-power ultrasound results in the formation of some new compounds such as 2-methylbutanol, Methylisobutyl ketone, and 1-propene-3-methylthio. Comparative analysis revealed that the contents of the potatoes flavor compounds in the UIF-150 group exhibited closer alignment with those of the control group. Additionally, in region 2, the content of volatile substances decreased significantly under UIF-450 treatment, which might be because high-power ultrasound increased damage to the potato cell structure and promoted water migration during freezing. Along with the dissolution and release of volatile substances, the content of volatile flavour compounds decreased.

### 3.5 Effect of different freezing methods on the microstructure

#### 3.5.1 SEM analysis

Through microstructure to characterise and observe the mechanical damage caused by ice crystal formation during freezing, the impact of freezing on the structure of food can be effectively evaluated [5]. The formation of large ice crystals during the freezing process increases mechanical damage to the structure of cells and tissues and severely destroys the integrity of cells and tissues [61].

**Fig. 6:**
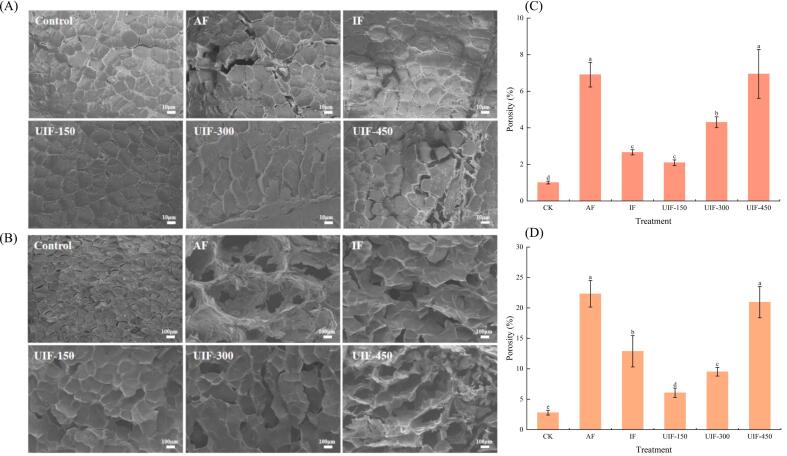
Microstructure of beef (A, magnification: 300×) and potatoes (B, magnification: 50×) and porosity of beef (C) and potatoes (D) in braised beef with potato dishes under different freezing treatments. Different lowercase letters (a–e) indicate a significant difference (*P* < 0.05) between samples. AF, air freezing; IF, immersion freezing; UIF, ultrasound-assisted immersion freezing at different ultrasound powers (150, 300 and 450 W).

As shown in Fig. 6A, the microstructure of beef indicates that different freezing treatments have caused varying degrees of damage to the muscle fibre structure. Compared with the control sample, the freezing treatment resulted in an increase in spacing between muscle fibres and varying degrees of tearing and fragmentation of the intrafibrillar membrane. The damage is primarily attributed to the ice crystals generated during the freezing process that squeeze and destroy the microstructure of muscle fibres, leading to muscle fibre rupture and structural damage [33]. Results indicated that the muscle fibre structure was damaged most severely in the AF treatment, with the muscle fibre bundles dispersed, resulting in an increase in the spacing between muscle fibres and the formation of larger pores. This might be due to the slow freezing process, which is particularly prone to the formation of large, uneven ice crystals, thus leading to increased damage to the fibre structure. Cheng et al. found that compared with freezing at −20 °C, freezing at −40 °C resulted in a faster freezing rate and the formation of a larger number of small ice crystals, reducing cell and muscle damage and better preserving the perch fillets [41]. UIF can effectively alleviate the damage to food quality caused by ice crystal formation during freezing. Results indicated that the beef muscle fibre structure was the most complete in the UIF-150 treatment among other treatments, with the fibre bundles arranged neatly and closely, resulting in smaller fibre gaps. Meanwhile, as shown in Fig. 6C, the muscle fibre porosity of beef in the UIF-150 group was the lowest after freezing. Compared with the AF group, the muscle fibre porosity of beef in the UIF-150 group decreased by 69.69 %, which may be attributed to the significant increase in the freezing rate of dishes by UIF, shortening the time of passing through the maximum ice crystal formation stage and effectively inhibiting the formation of large ice crystals. Additionally, cavitation generated by ultrasound can promote the formation of crystal nuclei and can better regulate the formation and distribution of fine ice crystals [36]. However, an increase in ultrasonic power to some extent increased the damage to the structure and texture of dishes. Findings revealed that the beef muscle fibre structure in the UIF-450 group experienced pronounced disassociation, distortion and fracturing, accompanied by the formation of substantial inter-fibre voids. Similarly, Sun et al. [62] discovered that excessive ultrasonic power would tend to promote the growth of larger ice crystals, resulting in a decrease in carp quality. These results can be attributed to the heightened heat generation caused by excessive ultrasonic power, the significant decrease in freezing rate, the rupture of the cavitation bubble and mechanical vibration generated by bubble propagation that led to increasing damage [39,51].

As shown in Fig. 6B, the cell morphology of the potatoes in the control group remained intact, with cells tightly arranged and almost no visible pores. By contrast, the cells of the potatoes in the AF group exhibited separation and deformation, with tissues severely collapsed and shrunken tissues, and larger pores appeared in the interior. Compared with the other freezing treatments, the potato cells in the UIF-150 group exhibited clear contours, compact and complete structure, and smaller pores, resembling more closely the control group in terms of microstructural integrity. As the ultrasonic power increased, the potato cells began to separate and the damage to the cell structure progressively intensified. The cell structure of potatoes in the UIF-300 group was damaged, with obvious holes appearing. The cells of potatoes in the UIF-450 group were severely deformed, with cell shrinkage, stacking and structural collapse, which led to the formation of large holes, akin to the results observed in beef. As shown in Fig. 6D, the porosity of the potato cells increased with an increase in ultrasonic power, and the porosity value of potatoes in the UIF-450 group increased by 71.07 % compared with that in the UIF-150 group. Results indicated that excessive ultrasonic power seriously damaged the tissue structure of potato cells, resulting in an increase in the number and size of pores in the cells. Therefore, UIF-150 freezing treatment can effectively alleviate the negative effects caused by ice crystal growth, thereby better maintaining dish quality.

#### 3.5.2 TEM analysis

**Fig. 7:**
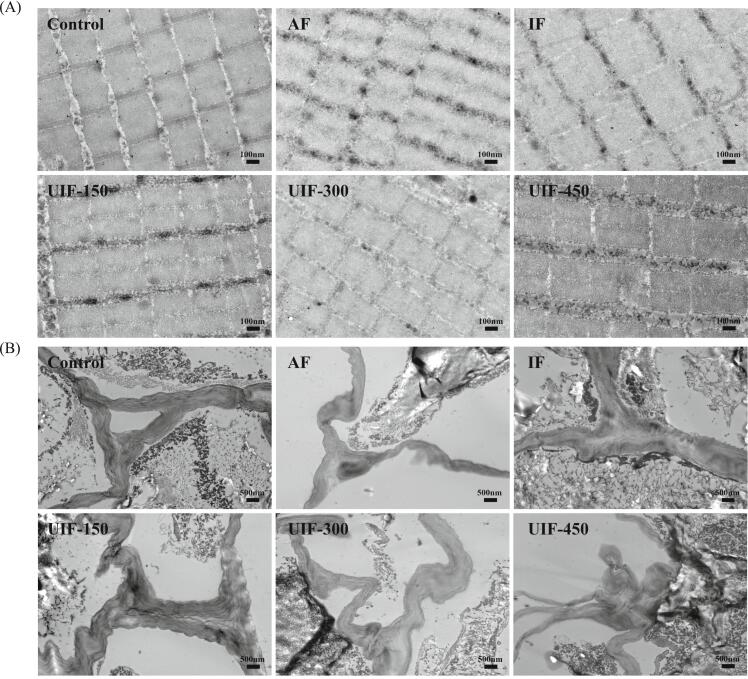
Ultrastructure of beef (A, magnification: 12000×) and potatoes (B, magnification: 3000×) in braised beef with potato dishes under different freezing treatments. AF, air freezing; IF, immersion freezing; UIF, ultrasound-assisted immersion freezing at different ultrasound powers (150, 300 and 450 W).

The ultrastructure can reflect changes in fibre and cell structures of food tissues and is an effective method for assessing quality [29]. As shown in Fig. 7A, compared with the unfrozen samples, the ultrastructure of beef indicated that freezing treatment resulted in varying degrees of damage to the muscle fibres. The most notable damage to the myofibrils was observed in the AF group, where the myofibrils exhibited severe warping and deformation, fractures occurred, the bright bands in the fibres became blurred and the Z line disappeared. This could be attributed to the extrusion damage to the myofibrils by the formation of ice crystals. These findings corroborate the previous ultrastructural observations by Lin et al. [2], who documented analogous myofibrillar warping and transverse fracture phenomena resulting from ice crystal formation. Compared with the AF group, the degree of myofibrillar distortion of beef in the IF group was reduced; however, the longitudinal sarcomere lengths in the fibres were significantly larger, and the bright bands remained blurred. Results indicated that the muscle fibre structure of beef in the UIF-150 group remained relatively intact, with sarcomeres arranged orderly, bright bands clearly discernible and the Z line only slightly obscured. As the ultrasonic power increased, the integrity of the muscle myofibrils decreased and the muscle fibres became distorted. In the UIF-300 group, the muscle fibres were obviously squeezed and deformed, with an increased number of pores observed in the interior, and the bright band became less distinct. In the UIF-450 group, the muscle fibres appeared ruptured, the bright bands were no longer visible and the entire structure seemed to have fused together.

As shown in the ultrastructure of potatoes in Fig. 7B, the cell walls of potatoes in the control group were compact in texture and exhibited a triangular steady-state structure between adjacent cell walls, with air gaps formed. The cell walls were tightly adhered to the cells, maintaining structurally integrity. Results indicate that freezing treatment led to the destruction of the cell wall structure, resulting in twisting and deformation. Ice crystal extrusion caused the disappearance of air gaps between the cell walls and their separation from the cells. The hardness, toughness and rigidity of the plant foods are closely related to the strength and structure of the cell walls [5]. This is the primary reason for the decrease in the hardness value and soft texture of potatoes after freezing. The cell wall structure of potatoes remained relatively intact under UIF-150 treatment. An increase in ultrasonic power led to an augmented degree of distortion of the cell wall of potatoes, resulting in a more severe deterioration of the cell wall structure texture and a more distinct separation of cells from the cell wall, which is consistent with the results of the texture characteristics of potatoes.

## 4 Conclusions

This study explored effects of UIF on the quality, moisture distribution, flavour characteristics and microstructure of braised beef with potato dishes at different power levels (0, 150, 300 and 450 W). The feasibility and applicability of UIF in the freezing of prepared dishes were preliminarily evaluated. This study demonstrated that the UIF-150 treatment could effectively shorten the overall freezing time of beef and potatoes, enhancing the freezing rate, which is beneficial for preserving dish quality. Results of the colour, TPA, LF-NMR and microstructure analyses indicated that the UIF-150 treatment significantly reduced the damage caused by ice crystals to the structural integrity of beef muscle fibres and to the potato tissue and cell morphology. This treatment decreased water migration, preserved the overall colour of the dishes and better maintained the taste and texture of beef and potatoes. The assessment of the changes in volatile compounds in the dishes revealed that the contents of some flavour compounds were markedly reduced following freezing treatments. However, the UIF-150 treatment effectively retained flavour compounds, which might be due to its relatively higher freezing rate than other treatments. Nevertheless, the negative impacts on the texture preservation and flavour fidelity in the dishes with increasing ultrasonic power need to be considered and addressed. Therefore, the selection and optimisation of the ultrasonic parameters for various types of dishes should be considered in future research to adequately meet the needs of industrial dish processing and production.

## CRediT authorship contribution statement

**Liye Cui:** Writing – original draft, Methodology, Investigation, Conceptualization. **Hengxun Lin:** Methodology, Investigation. **Xiaojia Hu:** Resources, Formal analysis. **Wenqiang Guan:** Software, Methodology, Formal analysis. **Xia Li:** Writing – review & editing, Funding acquisition, Formal analysis. **Wei Jia:** Investigation. **Yiping Yang:** Software, Methodology, Formal analysis. **Yong Chen:** Methodology, Investigation. **Chunhui Zhang:** Supervision, Resources, Project administration, Funding acquisition.

## Declaration of competing interest

The authors declare that they have no known competing financial interests or personal relationships that could have appeared to influence the work reported in this paper.

## References

[1] Wang X., Jing P., Chen C., Wu J., Chen H., Jiao S. (2024). Research progress on microbial control techniques of prepared dishes. Food Phys..

[2] Lin H., Cui L., Chen Y., Yang Y., Chen X., Chisoro P., Li X., Blecker C., Zhang C. (2024). Integrating multiple microstructure and water distribution visual analysis to reveal the moisture release and quality deterioration of precooked beef during freezing-thawing-reheating processes. Food Chem..

[3] People's Network Research Institute China, Prepared dishes industry development report of the China. http://yjy.people.com.cn/n1/2023/0710/c440911-40031856.html, 2023 (accessed October 11, 2024).

[4] Jia Y., Hu L., Liu R., Yang W., Khalifa I., Bi J., Li Y., Zhen J., Wang B., Zhang Z., Zhang E., Li B. (2024). Innovations and challenges in the production of prepared dishes based on central kitchen engineering: A review and future perspectives. Innov. Food Sci. Emerg. Technol..

[5] Li D., Zhu Z., Sun D.-W. (2018). Effects of freezing on cell structure of fresh cellular food materials: A review. Trends Food Sci. Technol..

[6] Li X., Qian S., Song Y., Guo Y., Huang F., Han D., Zhang C., Blecker C. (2022). New insights into the mechanism of freeze-induced damage based on ice crystal morphology and exudate proteomics. Food Res. Int..

[7] Huang W., Wang X., Xia J., Li Y., Zhang L., Feng H., Zhang X. (2023). Flexible sensing enabled agri-food cold chain quality control: A review of mechanism analysis, emerging applications, and system integration. Trends Food Sci. Technol..

[8] Cheng L., Sun D.-W., Zhu Z., Zhang Z. (2017). Effects of high pressure freezing (HPF) on denaturation of natural actomyosin extracted from prawn (Metapenaeus ensis). Food Chem..

[9] Zhang S., Meenu M., Xiao T., Ren J., Hu L., Song T., Ramaswamy H.S., Yu Y. (2024). Changes in properties of myofibrillar protein and myofibrillar protein gel from freshwater fish after low-temperature and high-pressure collaborative treatment. Innov. Food Sci. Emerg. Technol..

[10] Wu G., Yang C., Bruce H.L., Roy B.C., Li X., Zhang C. (2023). Effects of alternating electric field assisted freezing-thawing-aging sequence on longissimus dorsi muscle microstructure and protein characteristics. Food Chem..

[11] Liu F., Yang N., Zhang L., Jin Y., Jin Z., Xu X. (2023). Effect of weak magnetic field on the water-holding properties, texture, and volatile compounds of pork and beef during frozen storage. Food Biosci..

[12] Manzocco L., Alongi M., Cortella G., Anese M. (2022). Optimizing radiofrequency assisted cryogenic freezing to improve meat microstructure and quality. J. Food Eng..

[13] Hafezparast-Moadab N., Hamdami N., Dalvi-Isfahan M., Farahnaky A. (2018). Effects of radiofrequency-assisted freezing on microstructure and quality of rainbow trout (*Oncorhynchus mykiss*) fillet. Innov. Food Sci. Emerg. Technol..

[14] Xu W., Bao Y., Gou H., Xu B., Hong H., Gao R. (2024). Mitigation of mechanical damage and protein deterioration in giant river prawn (*Macrobrachium rosenbergii*) by multi-frequency ultrasound-assisted immersion freezing. Food Chem..

[15] Xu B., Zhang M., Bhandari B., Cheng X. (2014). Influence of power ultrasound on ice nucleation of radish cylinders during ultrasound-assisted immersion freezing. Int. J. Refring..

[16] Yu H., Mei J., Xie J. (2022). New ultrasonic assisted technology of freezing, cooling and thawing in solid food processing: A review. Ultrason. Sonochem..

[17] Bhargava N., Mor R.S., Kumar K., Sharanagat V.S. (2021). Advances in application of ultrasound in food processing: A review. Ultrason. Sonochem..

[18] Cheng X., Zhang M., Xu B., Adhikari B., Sun J. (2015). The principles of ultrasound and its application in freezing related processes of food materials: A review. Ultrason. Sonochem..

[19] Zhang Z., Sun D., Zhu Z., Cheng L. (2015). Enhancement of crystallization processes by power ultrasound: Current state‐of‐the‐art and research advances. Comp. Rev. Food Sci. Food Safe..

[20] Islam M.N., Zhang M., Fang Z., Sun J. (2015). Direct contact ultrasound assisted freezing of mushroom (*Agaricus bisporus*): Growth and size distribution of ice crystals. Int. J. Refring..

[21] Fu X., Belwal T., Cravotto G., Luo Z. (2020). Sono-physical and sono-chemical effects of ultrasound: Primary applications in extraction and freezing operations and influence on food components. Ultrason. Sonochem..

[22] Zhang M., Haili N., Chen Q., Xia X., Kong B. (2018). Influence of ultrasound-assisted immersion freezing on the freezing rate and quality of porcine longissimus muscles. Meat Sci..

[23] Zhang C., Li Y., Xia X., Sun Q., Sun F., Kong B. (2023). Changes in protein oxidation, structure, and thermal stability of chicken breast subjected to ultrasound-assisted immersion freezing during frozen storage. Food Chem..

[24] Sun Q., Sun F., Xia X., Xu H., Kong B. (2019). The comparison of ultrasound-assisted immersion freezing, air freezing and immersion freezing on the muscle quality and physicochemical properties of common carp (*Cyprinus carpio*) during freezing storage. Ultrason. Sonochem..

[25] Tian Y., Zhang P., Zhu Z., Sun D.-W. (2020). Development of a single/dual-frequency orthogonal ultrasound-assisted rapid freezing technique and its effects on quality attributes of frozen potatoes. J. Food Eng..

[26] Li J., Han D., Huang F., Zhang C. (2023). Effect of reheating methods on eating quality, oxidation and flavor characteristics of Braised beef with potatoes dish. Int. J. Gastron Food Sic..

[27] Yu H., Xie J. (2023). Effect of different orthogonal double frequency ultrasonic assisted freezing on the quality of sea bass. Food Chem.: X.

[28] Holman B.W.B., Bailes K.L., Cork S.D., Hopkins D.L. (2025). The effect of sustainable vacuum packaging selection on the quality, colour stability, and freshness of lamb meat stored chilled for up to 20 weeks. Meat Sci..

[29] Luo Y., Bi Y., Du R., Yuan H., Hou Y., Luo R. (2023). The impact of freezing methods on the quality, moisture distribution, microstructure, and flavor profile of hand-grabbed mutton during long-term frozen storage. Food Res. Int..

[30] Lin H., He X., Liu C., Meng J., Guan W., Hou C., Zhang C., Wang W. (2022). Static magnetic field-assisted supercooling preservation enhances water-holding capacity of beef during subzero storage. Innov. Food Sci. Emerg. Technol..

[31] Sun X., Yu Y., Saleh A.S.M., Yang X., Ma J., Gao Z., Zhang D., Li W., Wang Z. (2023). Characterization of aroma profiles of chinese four most famous traditional red-cooked chickens using GC–MS, GC-IMS, and E-nose. Food Res. Int..

[32] Cheng H., Bian C., Chu Y., Mei J., Xie J. (2022). Effects of dual-frequency ultrasound-assisted thawing technology on thawing rate, quality properties, and microstructure of large yellow croaker (*Pseudosciaena crocea*). Foods.

[33] Zhou J., Sun Q., Wei S., Wang Z., Xia Q., Han Z., Liu Y., Liu S. (2023). Changes in microstructure, quality and water distribution of golden pompano (*Trachinotus ovatus*) muscles subjected to magnetic field-assisted immersion freezing during long-term frozen storage. J. Food Eng..

[34] Zhang L., Wang P., Sun X., Chen F., Lai S., Yang H. (2020). Calcium permeation property and firmness change of cherry tomatoes under ultrasound combined with calcium lactate treatment. Ultrason. Sonochem..

[35] Zheng L., Sun D.-W. (2006). Innovative applications of power ultrasound during food freezing processes—a review. Trends Food Sci. Technol..

[36] Xu B., Zhang M., Bhandari B., Cheng X., Sun J. (2015). Effect of ultrasound immersion freezing on the quality attributes and water distributions of wrapped red radish. Food Bioprocess Technol..

[37] Wei Q., Sun Q., Dong X., Kong B., Ji H., Liu S. (2024). Effect of static magnetic field-assisted freezing at different temperatures on muscle quality of pacific white shrimp (*Litopenaeus vannamei*). Food Chem..

[38] Soltani Firouz M., Sardari H., Alikhani Chamgordani P., Behjati M. (2022). Power ultrasound in the meat industry (freezing, cooking and fermentation): Mechanisms, advances and challenges. Ultrason. Sonochem..

[39] Zhang C., Sun Q., Chen Q., Kong B., Diao X. (2020). Effects of ultrasound-assisted immersion freezing on the muscle quality and physicochemical properties of chicken breast. Int. J. Refring..

[40] Guo Z., Ge X., Yang L., Ma G., Ma J., Yu Q., Han L. (2021). Ultrasound-assisted thawing of frozen white yak meat: Effects on thawing rate, meat quality, nutrients, and microstructure. Ultrason. Sonochem..

[41] Chen X., Liu H., Li X., Wei Y., Li J. (2022). Effect of ultrasonic-assisted immersion freezing and quick-freezing on quality of sea bass during frozen storage. LWT.

[42] Tian Y., Chen Z., Zhu Z., Sun D.-W. (2020). Effects of tissue pre-degassing followed by ultrasound-assisted freezing on freezing efficiency and quality attributes of radishes. Ultrason. Sonochem..

[43] Ma X., Mei J., Xie J. (2021). Effects of multi-frequency ultrasound on the freezing rates, quality properties and structural characteristics of cultured large yellow croaker (*Larimichthys crocea*). Ultrason. Sonochem..

[44] McDonnell C.K., Allen P., Duggan E., Arimi J.M., Casey E., Duane G., Lyng J.G. (2013). The effect of salt and fibre direction on water dynamics, distribution and mobility in pork muscle: A low field NMR study. Meat Sci..

[45] Wang H., Wang R., Song Y., Kamal T., Lv Y., Zhu B., Tao X., Tan M. (2018). A fast and non-destructive LF-NMR and MRI method to discriminate adulterated shrimp. J. Food Meas Charact..

[46] Wang B., Li F., Pan N., Kong B., Xia X. (2021). Effect of ice structuring protein on the quality of quick-frozen patties subjected to multiple freeze-thaw cycles. Meat Sci..

[47] Qian S., Hu F., Mehmood W., Li X., Zhang C., Blecker C. (2022). The rise of thawing drip: Freezing rate effects on ice crystallization and myowater dynamics changes. Food Chem..

[48] Pan N., Dong C., Du X., Kong B., Sun J., Xia X. (2021). Effect of freeze-thaw cycles on the quality of quick-frozen pork patty with different fat content by consumer assessment and instrument-based detection. Meat Sci..

[49] Wu Z., Ma W., Xian Z., Liu Q., Hui A., Zhang W. (2021). The impact of quick-freezing methods on the quality, moisture distribution and microstructure of prepared ground pork during storage duration. Ultrason. Sonochem..

[50] Zhang M., Xia X., Liu Q., Chen Q., Kong B. (2019). Changes in microstructure, quality and water distribution of porcine longissimus muscles subjected to ultrasound-assisted immersion freezing during frozen storage. Meat Sci..

[51] Sun Q., Chen Q., Xia X., Kong B., Diao X. (2019). Effects of ultrasound-assisted freezing at different power levels on the structure and thermal stability of common carp (*Cyprinus carpio*) proteins. Ultrason. Sonochem..

[52] Wang X.-Y., Xie J. (2019). Evaluation of water dynamics and protein changes in bigeye tuna (*Thunnus obesus*) during cold storage. LWT.

[53] Yang W., Dong Y., Ma X., Xie J., Mei J. (2024). Effects of multi-frequency ultrasound-assisted immersion freezing processing on myofibrillar protein structure and lipid oxidation of large yellow croaker (*Larimichthys crocea*) during long-time frozen storage. Ultrason. Sonochem..

[54] Li C., Zou Y., Liao G., Zheng Z., Chen G., Zhong Y., Wang G. (2024). Identification of characteristic flavor compounds and small molecule metabolites during the ripening process of Nuodeng ham by GC-IMS, GC–MS combined with metabolomics. Food Chem..

[55] Cárcel J.A., Benedito J., Bon J., Mulet A. (2007). High intensity ultrasound effects on meat brining. Meat Sci..

[56] Wang K., Arntfield S.D. (2014). Binding of carbonyl flavours to canola, pea and wheat proteins using GC/MS approach. Food Chem..

[57] Martínez-Arellano I., Flores M., Toldrá F. (2016). The ability of peptide extracts obtained at different dry cured ham ripening stages to bind aroma compounds. Food Chem..

[58] Wang T., Han D., Zhao L., Huang F., Yang P., Zhang C. (2023). Binding of selected aroma compounds to myofibrillar protein, sarcoplasmic protein, and collagen during thermal treatment: role of conformational changes and degradation of proteins. J. Agric. Food Chem..

[59] Guo Z., Teng F., Huang Z., Lv B., Lv X., Babich O., Yu W., Li Y., Wang Z., Jiang L. (2020). Effects of material characteristics on the structural characteristics and flavor substances retention of meat analogs. Food Hydrocoll..

[60] Zhang M., Li F., Diao X., Kong B., Xia X. (2017). Moisture migration, microstructure damage and protein structure changes in porcine longissimus muscle as influenced by multiple freeze-thaw cycles. Meat Sci..

[61] Zhou P., Chu Y., Lv Y., Xie J. (2022). Quality of frozen mackerel during storage as processed by different freezing methods. Int. J. Prop..

[62] Sun Q., Zhao X., Zhang C., Xia X., Sun F., Kong B. (2019). Ultrasound-assisted immersion freezing accelerates the freezing process and improves the quality of common carp (*Cyprinus carpio*) at different power levels. LWT.

